# Minimal residual disease assessment of papillary thyroid carcinoma through circulating tumor cell‐based cytology

**DOI:** 10.1002/cam4.4813

**Published:** 2022-05-18

**Authors:** Nadia Innaro, Rita Gervasi, Teresa Ferrazzo, Nastassia C. Garo, Lucia S. Curto, Annamaria Lavecchia, Isabella Aquila, Giuseppe Donato, Natalia Malara

**Affiliations:** ^1^ Unit of Endocrinological surgery Mater Domini Hospital of Catanzaro Catanzaro Italy; ^2^ Department of Medical and Surgical Science University “Magna Græcia” Catanzaro Catanzaro Italy; ^3^ Department of Health Sciences University “Magna Græcia” Catanzaro Catanzaro Italy; ^4^ Pathology Unit Pugliese Hospital Catanzaro Italy; ^5^ BioNEM Laboratory and Nanotechnology Research Center, Department of Experimental and Clinical Medicine University "Magna Graecia" of Catanzaro Catanzaro Italy

**Keywords:** circulating tumor cells, cytology CTCs, Liquid biopsy, PTC, recurrence risk, short‐time cultured CTCs

## Abstract

The overall estimated risk of recurrence after an apparently complete thyroid cancer resection ranges from <1% to 55%, and the high‐quality pathology report is crucial for proper risk stratification. The neck ultrasound (US) and serum thyroglobulin (Tg) and anti‐Tg antibody (TgAb) assays are the mainstays for Differentiated Thyroid Cancer (DTC) follow‐up. However, the neck US includes a high frequency of nonspecific findings and despite the serum, Tg unmasks the presence of thyrocytes, it is not discriminating between normal and malignant cells. In this study, to improve post‐surgery follow‐up of minimal residual disease in papillary thyroid cancer (PTC) patients, blood‐derived cytology specimens were evaluated for the presence of circulating tumor cells (CTCs). The presence of CTCs of thyroid origin was confirmed by cytomorphological and tissue‐specific antigens analysis (Thyroid Transcription Factor‐1/TTF‐1 and Tg) and proliferative profile (percentage of cells in S‐phase). Our data revealed an unfavorable’ prognostic risk in patients with >5% CTCs (*p* = 0.09) and with >30% S‐phase cells at baseline (*p* = 0.0015), predicting ≤1 year relapsing lesion event. These results suggest a new intriguing frontier of precision oncology forefront cytology‐based liquid biopsy.

## Graphical abstract

minimal residual disease assessment in post surgery patients with papillary thyroid cancer through CTCs‐based cytology

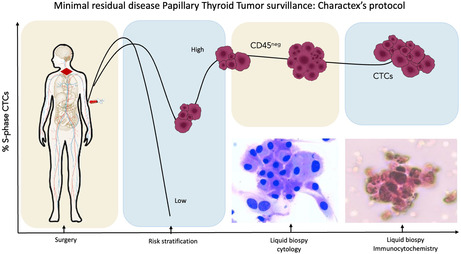

**Abbreviations**
CTC: Circulating tumor cells
TC: Thyroid cancer
PDPN: Podoplanin
TSHR: TSH receptor
OS: Overall survival
DFS: Disease‐free survival
CHARACTEX: CHARActerization of Circulating Tumor cells and Expansion
ICD: Informed consent document
HS: Healthy subjects
CP: Cancer patients
FNAC: Fine‐needle aspiration cytology
DTC: Differentiated thyroid cancer
PTC: Papillary thyroid cancer
PBS: Phosphate Buffered Saline
BDC: Blood‐derived culture
DTC: Differentiated Thyroid Cancer
DM: Distant Metastasis

## 1 INTRODUCTION

Among differentiated thyroid cancers (DTC) the papillary thyroid cancers (PTCs) are the most common histological subtypes. They have increased in incidence and mortality rate in the last years.^1^, ^2^ In 2015, the American Thyroid Association individuated criteria to estimate the risk of recurrence after apparent complete resection. The overall estimated risk of recurrence ranges from <1% to 55% and is classified as low (<5%), intermediate (6%–20%), or high (>20%). The initial risk class assignment is strictly dependent on the high‐quality pathology and is revised during the follow‐up relatively to the evolution of the disease and the response to therapy (dynamic risk stratification).^3^ According to the literature, several risk factors for recurrence in persistent/recurrent PTC have been recognized, such as age at surgery treatment, aggressive histology, location, and size of recurrent lesions.^4^ The follow‐up based on biochemical and imaging comprises prevalently serum thyroglobulin (Tg) levels and neck ultrasonography (US). Serum Tg is a sensitive marker revealing the presence of thyrocytes but is unable to discriminate between normal and malignant cells. Its undetectable levels correspond to high negative predictive values, however, if detectable, they can be false positive for the presence of cancer cells. Moreover, concomitant assessment of serum antibodies against Tg (TgAb) is mandatory, because these antibodies can interfere with Tg tests, causing false results.^5^ Neck US is the most effective imaging investigation to define structural disease in the neck. In the preoperative phase, cytology and serum Tg detection, in combination with fine‐needle aspiration (FNA), can lead to almost 100% accuracy.^1^ In the postoperative phase, unmasking PTC metastasis involving locoregional lymph nodes is highly dependent on the neck US operator, because of a high frequency of nonspecific findings and the possibility of unsatisfactory visualization of deep structures including those acoustically shaded by bones or air.^4^ In this study, we contribute to improving the dynamic risk stratification in postoperative PTC‐patients, by analyzing circulating tumor cells (CTCs), alongside biochemical and structural results by using serum Tg levels and US neck analysis, as indicated by guidelines. CTCs are malignant cells disseminated from the primary tumor mass which migrate into the bloodstream and may be able to develop distant metastases. The post‐surgery detection of these cells in patients surgically treated for PTCs denounces and defines the minimal residual disease (MRD). Although the goal of surgical therapy is to eradicate all malignant cells, a substantial portion of patients shows the subclinical detectable value of cancer cells, so‐called MRD that ultimately leads to relapse. A critical issue in MRD management is its quantification by multiple and repetitive measurements to detect cutoff corresponding to the minimal threshold of still detectable cancer cells to indicate. Clinical practice around the world varies and no gold standard method was identified for quantifying MRD to orient the therapy toward a more or less aggressive treatment.

Several studies have shown that the identification and quantification of circulating tumor cells can be used as prognostic markers of survival and MRD^6^ in various solid tumors, such as colon, prostate, and breast.^7^, ^8^, ^9^ Currently, there is one FDA‐approved method for CTC enumeration (Cell Search). More recently, the focus has shifted to CTC characterization which holds great promises for predictive testing.^10^ In this way, here we report our experience by using Malara's protocol to isolate and short‐time expand circulating tumor cells in thyroid cancer patients, as previously reported. ^11^, ^12^, ^13^, ^14^ The short‐time expansion of CTCs was used with a triple aim: 1‐ making evident the rare population of CTCs through their direct expansion; 2‐ analyzing their proliferation phase to obtain prognostic information useful to improve patient management; 3‐ making cytomorphological evaluation supported by ancillary methods such as immunocytochemical assays on expanded CTCs like in a traditional cytopathological approach permitting us to characterize the MRD by a noninvasive way.

## 2 MATERIALS AND METHODS

### 2.1 Patients selection

Blood samples have been collected from healthy subjects and thyroid cancer patient volunteers of the Unit of Endocrinological Surgery Mater Domini of Catanzaro, from July 2013 up to July 2020. The project entitled CHARACTEX (CHARActerization of Circulating Tumor cells and Expansion) has been approved by the local institutional review board and conducted according to the recommendations of the Declaration of Helsinki and its amendments. The approval number of the CHARACTEX project is 2013.34. All patients enrolled in this study signed an informed consent document after having read and commented about the information on the project and related experimental protocol with the researcher in charge. The inclusion criteria adopted to enroll volunteers were:

Tumor patients.

- Caucasian race
- Patients with a diagnosis of PTC
- Age between 18 and 85 years
- Effective contraceptive methods in cases where there is the possibility of conception.
- Written informed consent.

Healthy subjects or control.

- Caucasian race
- Nonsmoking healthy male and female adults
- Age between 18 and 85 years
- State of good health supported by the most relevant clinical and biochemical parameters
- Effective contraceptive methods are used in cases where conception is possible.
- Written informed consent

### 2.2 Blood‐based cytology specimens

The peripheral blood was taken both from control and cancer patients. Peripheral blood samples were collected into EDTA tubes with a total volume of 7 ml. The expansion phase was performed through in vitro seeding of the cellular suspension isolated from the blood by using Malara's protocol^11^, ^12^, ^13^, ^14^ in chamber slides and on Petri dishes, for 14 days and periodically checking for cell growth and the need to add more medium. At the end of 14 days, the cells were fixed on slides and collected from a Petri dish for further characterization.

### 2.3 Cytometry analysis

Flow cytometry was performed with a FACS ARIA III (Becton Dickinson) analyzed with FACSDiva v. 6.1.3, and FACSuite v1.05 (BD) software. Data were analyzed using a multivariate mathematical approach to identify several cellular patterns across the different patients. Blood‐derived cultures performed on plates were analyzed with anti CD45 antibody (CD45, Clone 2D1 Becton Dickinson, Cat# 564327) designed to identify populations of naïve hematological cells. For the proliferation profile cells (3–7) × 10^5^ have collected and cell cycle phase's distribution was performed using the Becton Dickinson kit: CycleTEST plus DNA reagent Kit, data acquisition using FACS Canto II (Becton Dickinson), and the analysis was performed with ModFit LT software (http://modfit‐lt.software.informer.com/4.0/).

### 2.4 Blood‐based cytology specimens characterization

The slides were fixed and then stained by Diff‐Quick or Hematoxylin–Eosin method for cytomorphological evaluation established by pathologists experienced in cancer disease by a double‐blind procedure. Immunocytochemical staining was performed by using standard reagents and techniques, as previously described.^15^, ^16^ Thyroglobulin clone 1D4, dilution 1:50, and Thyroid Transcription Factor‐1 (TTF‐1) dilution 1:25. Staining was performed using a biotin‐free polymeric horseradish peroxidase‐linker antibody conjugate system, and the slides were visualized with 3, 3′‐diaminobenzidine (DAB) solution (1 mm DAB, 50 mm Tris–HCl buffer [pH 7.6], and 0.006% H2O2). In addition, the nuclei were counterstained with hematoxylin, and the slides were dehydrated through a series of graded alcohols (70%, 90%, and 100%), cleared in xylene (Sigma‐Aldrich, St. Louis, MO, USA), and mounted.

### 2.5 Statistical analysis

All statistical analyses were performed using MedCalc for Windows, version 18 (MedCalc Software, MariaKerke, Belgium). To analyze, the relationship between markers expressions, tumor characteristics, and clinical parameters Pearson correlation test was applied. Disease‐free survival analysis has been conducted using the Kaplan–Meier method, log‐rank test. Sub‐groups were compared through the Mann–Whitney analysis (for independent variables) with a valid statistical significance of *p* < 0.05. Each experimental data are expressed as mean and standard error (SE).^17^

## 3 RESULTS

### 3.1 Clinical characteristics of enrolled volunteers

Baseline characteristics of voluntaries enrolled are detailed in Table S1. Thyroid cancer patients and control subjects were enrolled in the prospective project CHARACTEX. Blood‐derived cell cultures were performed from peripheral blood of 20 volunteer oncologic patients (median age 38 years; 50% female and 50% male). Differentiated Thyroid Cancer (DTC) was diagnosed in all patients enrolled. In particular, all DTCs were PTCs among which 15% DTCs with distant metastasis and 85% were without. The extranodal invasion was revealed in 60% of DTCs. Multiple intra‐parenchymal tumor foci were found in 25% of DTCs. In particular, in 15% of metastatic DTCs and 10% of localized PTCs. Tumor size was <30 mm in 25% of DTCs. 70% of DTCs were stage I and II. In the post‐surgery, Tg levels were >1 ng/ml only in 15% of the enrolled DTCs. Moreover, 20 voluntary healthy subjects (median age 38 years; 50%female and 50% male) were involved in the study.

### 3.2 CTCs and proliferation profile

**FIGURE 1:**
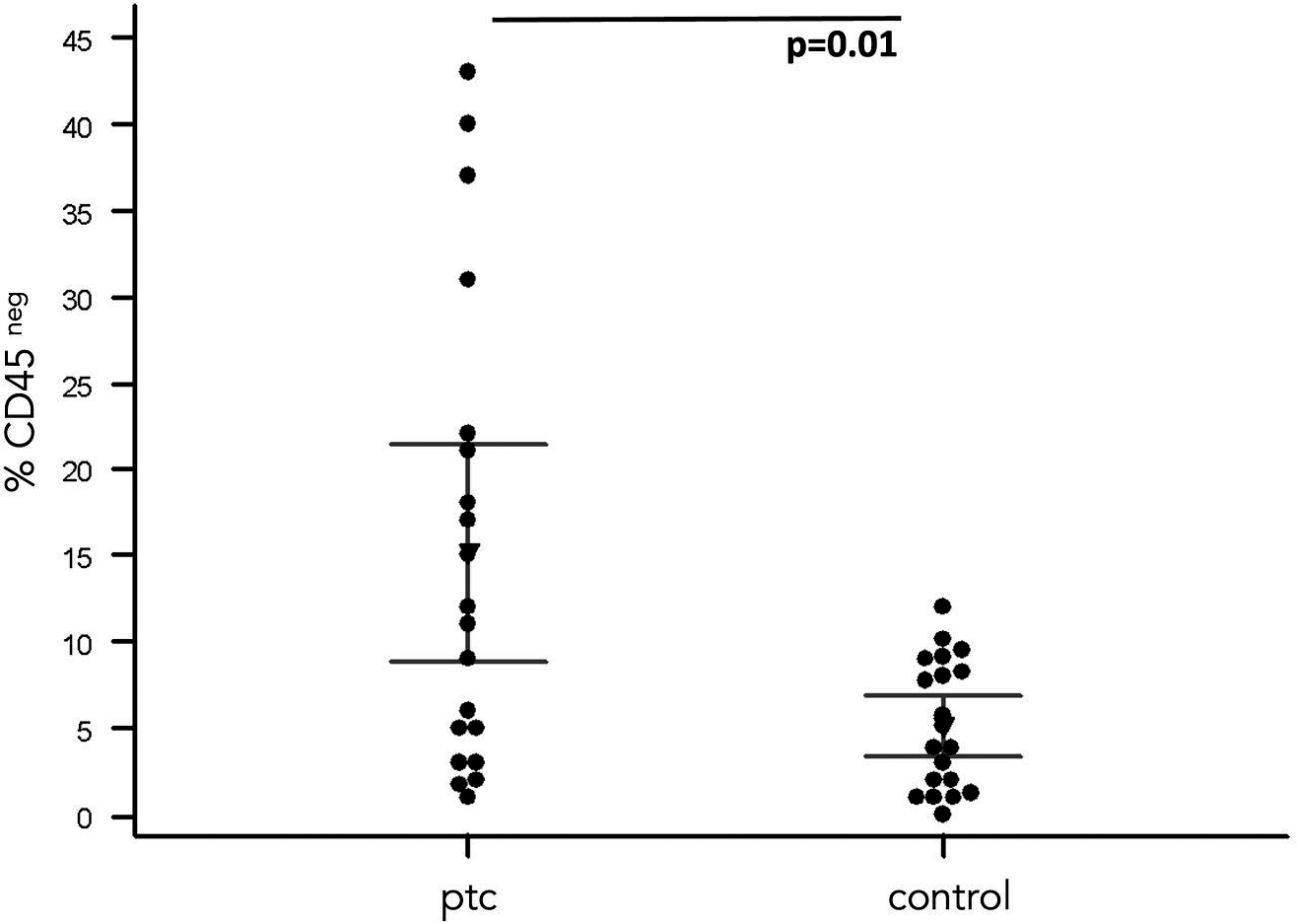
Percentage of cells negative for the expression of CD45 pan‐leucocytes antigen. The expression of the CD45 pan‐leucocytes antigen (% CD45neg) was investigated in cultured cells isolated for blood samples of papillary thyroid cancers (PTC) patients and healthy volunteers (control). Comparative analysis of the percentage of cells CD45neg shows a significant difference (*p* = 0.01) underlying the increased percentage of CD45neg cells in blood‐derived cultures of PTC––patients‐derived enriched for circulating tumor cells

**FIGURE 2:**
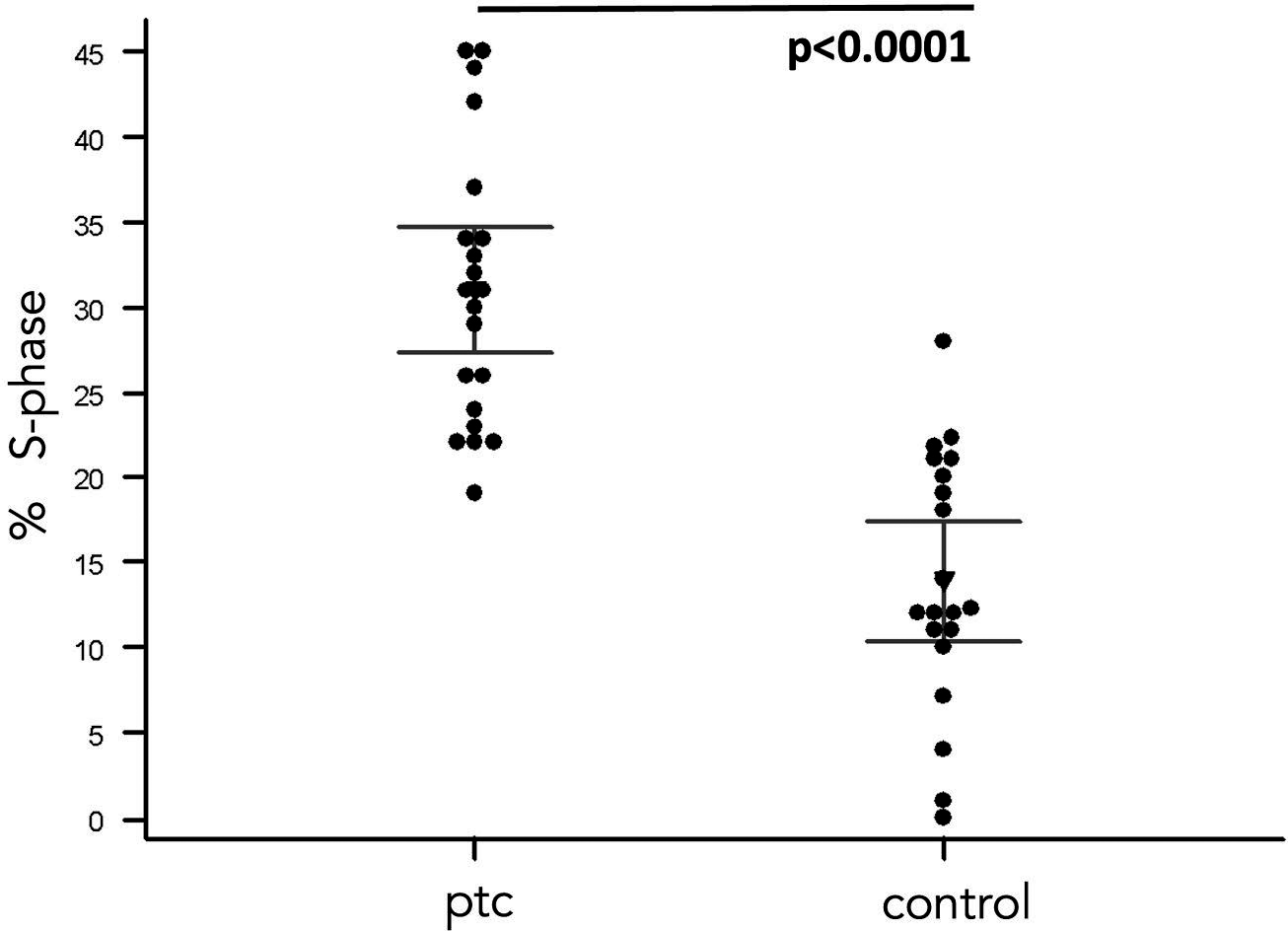
Comparative analysis of proliferating cells in blood‐derived cultures. Comparative analysis of the percentage of proliferating cells (% S‐phase) in blood‐derived cultures from papillary thyroid cancer (PTC) patients and healthy volunteers (control), shows a strongly significant difference (*p* < 0.0001) underlying the prevalence of circulating tumor cells in blood‐derived specimens of cancer patients

Flow cytometric analysis for CD45, pan leukocyte antigen, was used to highlight the non‐hematological component of the blood (CD45 negative cells) present at the end of 14 days in blood‐derived cultures (BDCs). The mean values of CD45 negative cells in the BDCs obtained from cancer patients showed 95% CI having a mean ranking of 8%–21% with a standard error of 2.9 and in the control group of 3%–6% with a standard error of 0.8. The difference between the two groups was significant (*p* < 0.01) (Figure 1). The percentage of proliferating cells (S‐phase cells) was analyzed through the cell cycle phase distribution. The PTC‐patients showed a percentage of S‐phase cells showed 95% CI having a mean ranging from 27 to 34% and standard error 1, 7. BDCs from the healthy cohort showed 95% CI having a mean ranging from 10% to 17% (1–6 was the standard error of the means). The comparative evaluation showed a strong difference in terms of proliferating cells between PTC‐patients and controls (*p* < 0.0001) highlighting the prevalence of proliferating cells (possible enrichment of CTCs) in BDCs from the cancer cohort (Figure 2).

### 3.3 Cytology and Immunostaining of CTCs

**FIGURE 3:**
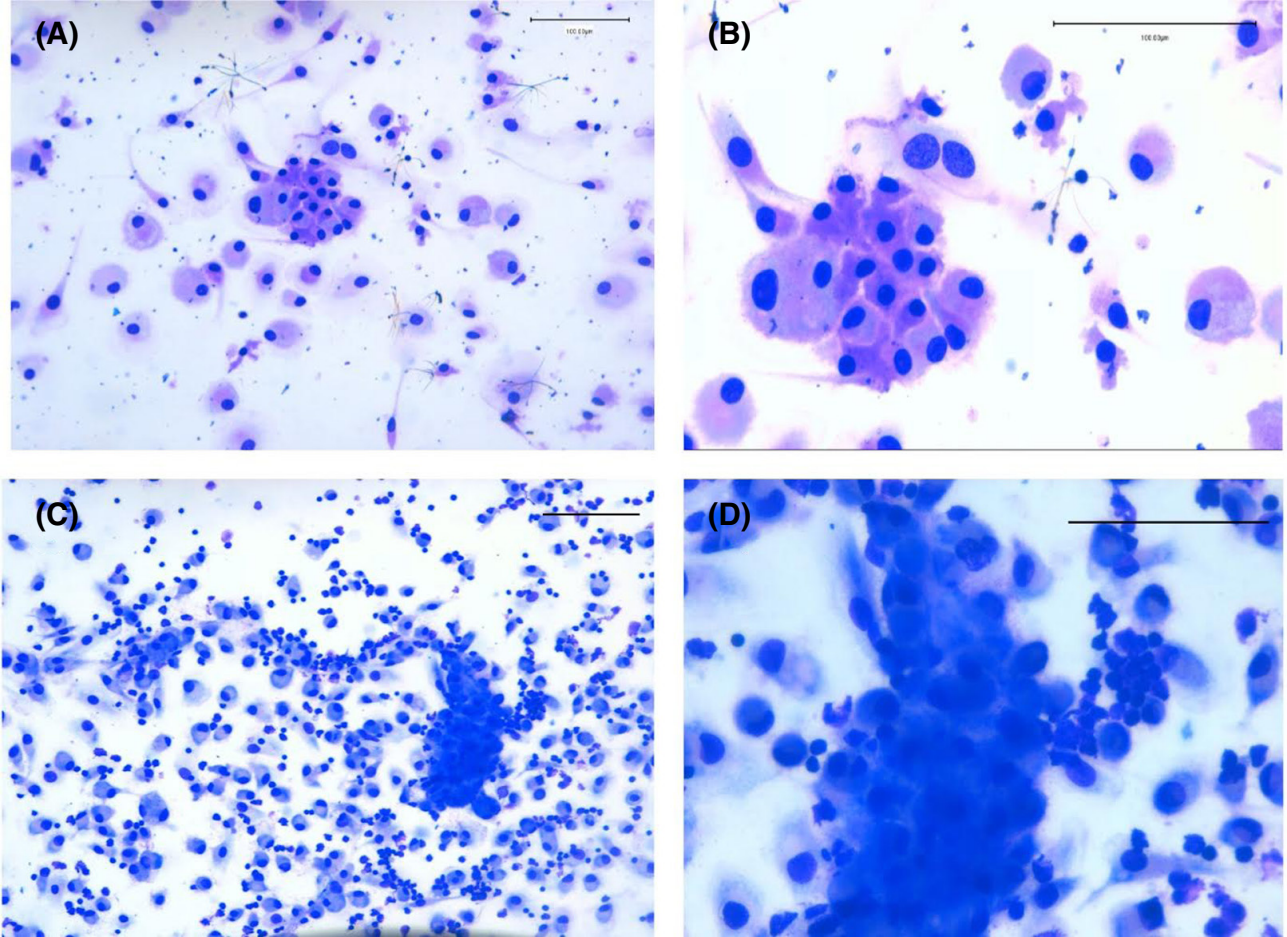
Cytology in Liquid biopsy. In (A) and (B) Diff‐quick staining of blood‐derived cytology specimens of postoperative PTC‐patient enriched for circulating tumor cells. Highly cellular composed of numerous monolayer sheets with a syncytial‐like appearance are typical of papillary thyroid cancer cells. (Scale bar, 100 μm)

**FIGURE 4:**
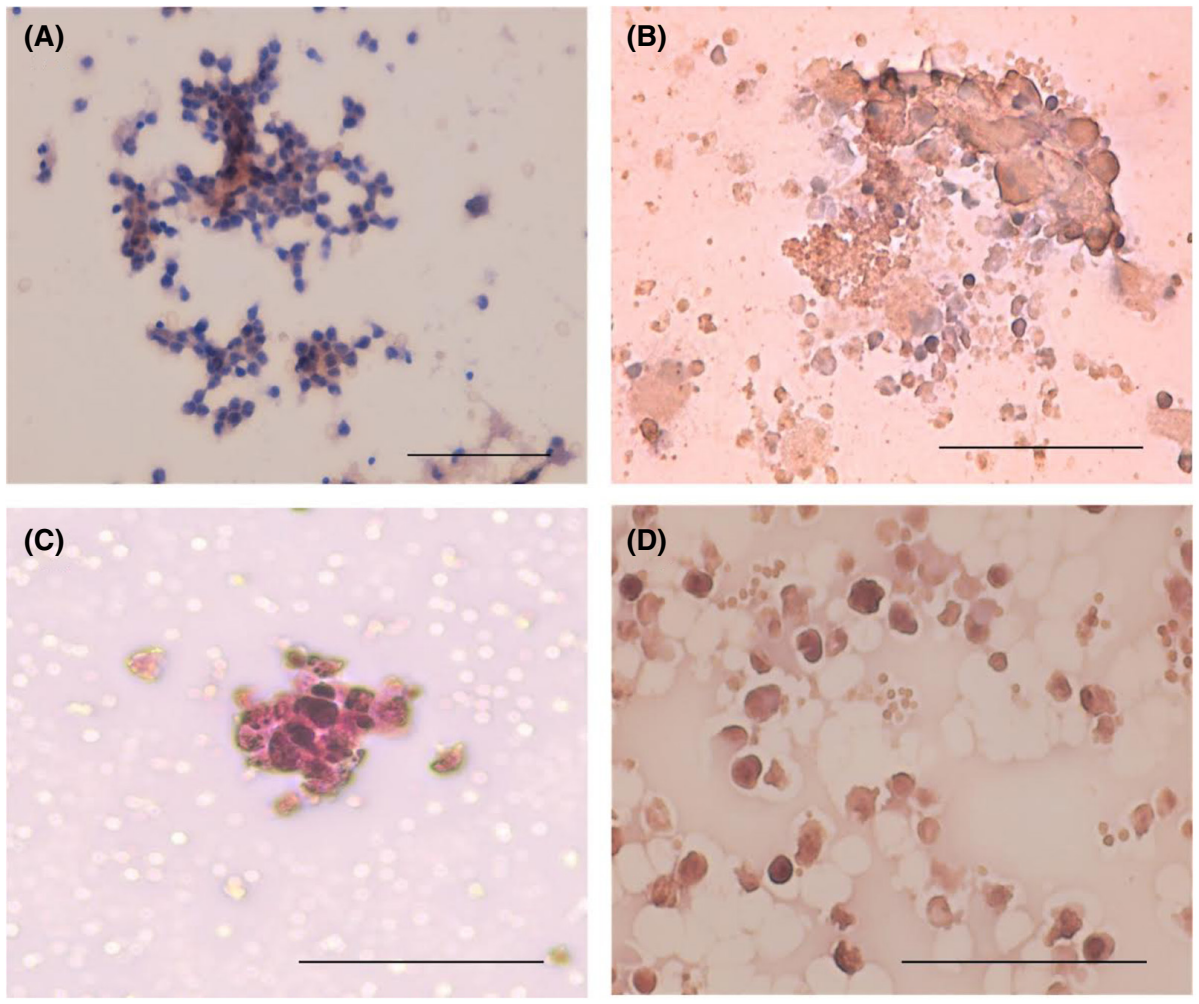
Immunocytochemistry on blood‐derived cytology preparations. In (A) and (B) immunochemical analysis for Thyroglobulin, in (C) and (D) for Thyroid Transcription Factor (TTF‐1) expression were performed. (Scale bar, 100 μm)

BDCs on chamber slides, 14 days old, were used for cytological evaluation. The cellular elements found in the blood‐derived cytological preparations obtained from PTC‐patients are characterized by the proliferation of medium‐sized elements with moderate atypia and a tendency to aggregate in structures with the papillary organization. Tumor cells are arranged in clusters or monolayer sheets but show less nuclear irregular contours or molding or crowding; nuclear atypia is more evident in the isolated elements Figure 3 (A and B) These cellular elements showed positivity for two specific markers of thyroid origin, nuclear TTF‐1, and cytoplasmic Tg on immunocytochemical analysis Figure 4(A–C).^18^ These findings confirm that the population of proliferating cells were composed of neoplastic thyrocytes.

### 3.4 Prognostic impact of CTCs in post‐surgery PTC‐patients

**FIGURE 5:**
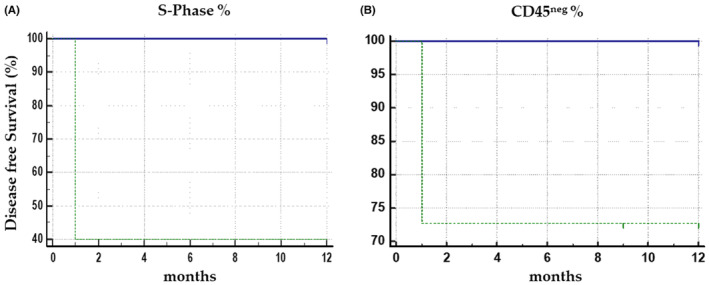
Preliminary results on disease‐free survival. In (A) Disease‐free survival (DFS) in thyroid cancer (TC)‐patients with S‐phase (green line >30%, blue line <30%) *p* = 0.001. In (B) DFS in TC‐patients with a percentage of cells CD45neg in blood‐derived cultures (green line >10%, blue line <10%) *p* = 0.09

Significant correlation between phase S > 30% and post‐surgical relapse or distant dissemination (DM) (*r* = 0.6784, *p* = 0.0010) was found in the PTC patient group. This data were further confirmed by the survival analysis here understood as disease‐free survival, shown in Figure 5. Furthermore, the intrathyroidal multifocality was recorded in the presurgical phase, both at the ultrasound examination and subsequently confirmed by the histological analysis after thyroidectomy correlation index side with the percentage of proliferating cells (*r* = 0.7316, *p* = 0.0002). The correlations of this functional parameter with the pre‐and post‐surgical phase of the disease, lymph node invasion, and tumor size were negative. Similarly, the percentage of CD45^neg^ cells presented in the BDCs of PTC‐ patients correlated with the traditional prognostic factors (extracapsular invasiveness, histological variant, grading, and multifocality). Statistical analysis shows a significant correlation between the percentage of CTCs (as a percentage of CD45^neg^ cells), only with post‐surgical recurrence (*r* = 0.4523, *p* = 0.0453). These data were also confirmed by the disease‐free survival analysis, Figure 5, which shows that PTC‐patients with a percentage of CD45^neg^ cells greater than 10% showed a greater risk of post‐surgical relapse.

## 4 DISCUSSION

The role of CTCs in thyroid cancer is still ongoing, with very few studies establishing the prognostic significance of CTCs in patients with thyroid cancer. In literature, the prognostic value significance of CTCs was evaluated by their quantification before and after surgical treatment of the primary lesion.^19^ In this study, the measurement of CTC levels occurred through the quantification of TSH receptor (TSHR)‐mRNA; reduction in CTC levels on the first postoperative day was present in disease‐free patients, but this condition was not observed in patients who have developed metastases or relapses. Therefore, a continuously high level of this marker after surgery could predict metastasis or recurrence.^19^ Winkens et al. assessed the number of CTCs in patients undergoing thyroidectomy; this value was significantly higher when compared to healthy controls. Furthermore, this study underlines the importance of intraindividual variation in the number of CTCs, detected by multiple dosages during postoperative follow‐up, rather than the value of a single CTC determination, thus attributing a predictive role to disease recurrence.^20^ Moreover, by using the Cell Search technology, CTC ≥5 was found to be an independent negative prognostic factor of overall survival (OS) in thyroid cancer patients. It was not possible to identify the prognostic or predictive role of CTCs in the PTC group due to the limitation of the Cell Search determination method based on the immunodetection of cell EpCAM positive.^21^ This limit is partially overcome by the adoption of an integrated strategy for the identification of CTCs through Cell Search and integrated immunostaining‐fluorescence. A correlation between the number of CTCs ≥5 and the presence of distant metastases was detected.^22^ Another method of determining CTCs was through the EpCAM receptor, podoplanin (PDPN), and the TSHR. It was found that the number of CTCs were statistically higher in patients who were not in remission than in the control group. It has been shown that the OS of patients with thyroid cancer is closely related to the levels of CTC in the blood.^23^ Despite the majority of the clinical prognostic studies showing that CTC count indicates prognosis, few studies were conducted on the accuracy of the MRD characterization model to predict the efficacy of anticancer treatment. In the group of PTC‐patients we studied, the MRD was revealed by highlighting the presence of non‐hematological proliferating elements within the short‐term blood‐derived cultured cells. Further cell assignment of thyroid origin was assessed by the analysis of related antigens like TTF‐1 and Tg. Blood‐derived cultured PTC cells retain morphological characteristics described in the “Bethesda system for reporting thyroid cytopathology”,^24^ the same that is relievable with traditional fine needle aspiration cytology in diagnostic procedures before surgery. The post‐surgery monitoring approach here reported fixes the critical milestone in the MRD management in PTC‐patients, that is its quantification and qualification by multiple and repetitive measurements of detectable cancer cells within the blood for assessing, cytological based, the potential to relapse. The deep analysis of MRD must include the characterization of the high‐risk disease features and in agreement with the procedure adopted in the early phase of PTC diagnosis, it cannot disregard by the Bethesda system for reporting thyroid cytopathology evaluation. The meaning of the persistence of MRD in post‐surgical PTC‐ patients is indicative of a high risk of relapse and at the same time detecting MRD may indicate that the surgery was not completely effective or that was incomplete despite its evident macroscopically complete resection. Moreover, MRD may be present after a surgical treatment because not all cancer cells were removed, or because one or more cancer cells were hidden in other sites. Clinical practice around the world varies and no gold standard method was identified for quantifying MRD.^25^ Our method appears to offer an early, immediate usable tool for patient management. The methodology applied for the isolation from the peripheral blood of a cell suspension which is then interrogated, for a short time, in vitro in terms of proliferation, is sensitive enough to highlight a population of non‐hematological cells (i.e., CD45 negative) even in the samples of blood isolated from the control group. This epiphenomenon is not exclusive to this method, because it has been reported in the literature that in healthy subjects it is possible to identify a circulating non‐hematological fraction that prevalently corresponds to the fraction of circulating endothelial cells.^26^ Conditions such as menstruation, sport, or altitude can modify the rate of availability of these cells in the peripheral blood, as already described.^27^ The fraction of CD45^neg^ cells in the peripheral blood of healthy subjects is therefore not a worrying condition. The methodology reported here shows that the discriminatory functional characteristic between the two populations of non‐hematological cells between the two groups, tumor and healthy, is prevalently represented by the S‐phase. The population of cells present in the blood‐derived cytological preparations obtained from the peripheral blood of PTC‐patients is characterized by a significantly higher percentage of proliferating cells than the healthy counterpart, as previously demonstrated also for other tumors.^28^ Moreover, the proliferative phase, the S‐phase, by what is considered in terms of tumor tissue proliferation, also for the population of circulating tumor cells is a functional marker that identifies them and at the same time allows to stratify the patient population according to a prognostic order faithful to the evolution and progression of the disease. On the other hand, extensive development of CTC enrichment technologies has reached a point at which the main challenge has shifted toward the molecular and genetic analysis of CTCs captured by different microelectromechanical systems over the last 20 years.^29^, ^30^, ^31^, ^32^, ^33^ Cytopathological identification of the tumor cells in the peripheral blood samples evaluated both cytomorphological through the cytological staining of the Diff‐quick, and through the immunocytochemistry approach, which can achieve an almost 100% accuracy in combination with neck US. The potential limitation of this methodology could be due to the presence of low proliferating tumor cells unable to make the rare population of CTCs evident through the short‐time in vitro passage.^17^, ^34^ Patients with a relapse consisting of distant metastases are here identified by real cancer cells residual population, even if at the subclinical phase. Moreover, sometimes suspicious sites of metastasis in operated patients are difficult to reach to have cytological or histological confirmation. In these cases, short‐term expansion of cultured CTCs could be a quick and effective solution to the diagnostic problem.

## 5 CONCLUSIONS

Despite this methodology having been extensively standardized, major requirements and guidelines for biological parameters are needed to be clinically approved. Besides its proven clinical relevance and cost‐effectiveness, this method responds to strict specificity and sensitivity criteria, is reproducible, easy‐to‐perform, and to interpret allowing to have evaluable CTCs for further molecular assay to improve the therapeutic options. In addition, it is also potentially available as a screening method that allows differentiating those rare cases considers doubtful at cytology assessment.

## CONFLICT OF INTEREST

None.

## Supporting information


Table S1

## Data Availability

The data that support the findings of this study are openly available ionline at http://www.bionem.unicz.it/web/.
